# Addressing context to understand physical activity among Muslim university students: the role of gender, family, and culture

**DOI:** 10.1186/s12889-019-7670-8

**Published:** 2019-11-05

**Authors:** Ghadir Fakhri Aljayyousi, Maher Abu Munshar, Farid Al-Salim, El Rayah Osman

**Affiliations:** 1 Department of Public Health, College of Health Sciences, Qatar University, PO Box 2713, Doha, Qatar; 2 Department of Humanities, College of Arts and Sciences, Qatar University, Doha, Qatar; 3 Department of International and Middle Eastern Studies, College of Arts and Sciences, The American University in Dubai, P.O. Box 28283, Dubai, UAE; 4 Department of Social Sciences, College of Arts and Sciences, Qatar University, Doha, Qatar

**Keywords:** Physical activity, Adolescence, Family, Religion, Culture

## Abstract

**Background:**

Physical inactivity is a crucial risk factor for the development of chronic health issues, which have a high incidence among Arabs living in the Gulf Cooperation Council (GCC) countries. The Qatar Stepwise Survey 2012 reported that approximately 44% of young adults 18–44 years of age had insufficient levels of physical activity. Family is a powerful source of information and socialization for adolescents and has a strong influence on their attitudes, decision-making, and behaviors.

**Methods:**

The purpose of this study is to understand how university students’ physical activity can be influenced by sociocultural factors, particularly family health values and Muslim Arab culture. Using the criterion sampling strategy, 20 undergraduate Muslim students (Female students =10, Male students = 10) aged from 18 to 23 years who were Qatari or born and also raised in Qatar were recruited and interviewed. Participants were asked if they consider themselves active or not, about their perception of family health values regarding physical activity and the factors shaping these values, and the influence of family values on their physical activity behavior. The interviews were transcribed verbatim, coded, and analyzed following inductive analysis.

**Results:**

The majority of the participants were influenced by their family health values, which were shaped by Qatari culture and the culture of origin for non-Qatari and were implicitly shaped by Islam. Participants reported that their role models of physical activity were males (fathers and male siblings), a health condition will motivate their families to be physically active***,*** and families give priority to work and academic achievement over physical activity. A few participants showed that there was explicit influence of Islam on their physical activity, because culture’s influence was veiling religion’s. Culture was seen as a facilitator for physical activity from the males’ perspectives, which was not the case for female participants who reported the negative influence of culture on their physical activity because of the limited choices available for them. Non-Qatari students revealed that their culture of origin (such as Syria, Palestine, Egypt, Somalia, Bangladesh, Sudan, Pakistan and India) was the dominant factor in shaping their family health values.

**Conclusions:**

The findings address gaps in the literature about families’ health values regarding physical activity in Qatar, the influence of the different ecologies surrounding these values, and the physical activity behaviors of university students. Knowledge about these factors can aid in the development of family-based interventions designed to motivate adolescents to be physically active, which should be religion- and culture-tailored.

## Background

Physical activity is defined as any bodily movement produced by skeletal muscles that results in energy expenditure [1]. For individuals aged 18–64 years, physical activity may include leisure time (e.g., walking, dancing, gardening, hiking, swimming), transportation (e.g., walking or cycling), occupational (i.e., work), household chores, play, games, sports and planned exercise in the context of daily, family, or community activities [2].

World Health Organization [3] reported that globally, more than 80% of the world’s adolescent population is insufficiently physically active and 1 in 4 adults is not active enough. Research conducted in countries of the Gulf Corporation Council (GCC) revealed that only 40% of men and 27% of women reported that they were physically active [4]. Additionally, the Qatar Stepwise Survey 2012 reported that approximately 44% of young adults 18–44 years of age had insufficient levels of physical activity with women had higher levels of low prevalence of physical activity than men (54.2% vs. 37.4%) [5].

Physical inactivity is a crucial risk factor for the development of many chronic health issues, including obesity, type 2 diabetes, cardiovascular diseases, and breast and colon cancer, which have a high incidence among Arabs living in the Middle East and the Gulf Cooperation Council (GCC) countries [6]. In Qatar, the STEPwsie Report 2012 results illustrate that cardiovascular diseases are the leading causes of mortality and morbidity, diabetes has a high prevalence affecting 16.7% of the population, and 70.1% of the studied population were classified as overweight with BMI equal or above 25 kg/m2. Breast cancer, a leading cancer in Qatari women [6] has also shown an association with physical inactivity [7]. The government in Qatar has been promoting and supporting the active lifestyle for the population [8]. The government encouraged recreational facilities that enhance engagement in physical activity. In 2012, the ruler in Qatar announced 14 February as National Sports Day and people in Qatar celebrate this day as a national holiday and participate in variety of sports and activities. Yet, the prevalence of physical inactivity in the country is alarming.

Research has demonstrated that adolescents’ physical activity is shaped by sociocultural and environmental factors [9]. In Qatar, family is an important and powerful source of health information for adolescents [10]. Consequently, family would have a strong influence on their attitudes, decision-making, and behaviors. In a literature review about barriers and facilitators influencing the physical activity of Arabic adults, Benjamin and Donnelly [11] reported that lack of social support provided by families can be a barrier to physical activity. On the other hand, Muslim religion and having a good social support are among the factors that promote physical activity. Another study reported that having family members’ support would encourage Qatari women with heart issues to engage in healthy lifestyles, including physical activity [12].

In the Qatari context, as in modernized economies, people are wealthier, but there is still very much a close-knit, tribal, honor-oriented society. Qatari culture is religious and family-centered. Incorporating the family context will help in examining the role that social determinants of health embedded in families play in shaping the physical activity of adolescents. Research at the family level would help in examining cultural factors and other contexts to understand the phenomenon. To understand the dynamics of the family environment and how it may influence the physical activity of adolescents, it is vital to explore the role family values traditions play in shaping physical activity.

### Family values

Family values are usually multidimensional and are shaped by other ecological factors that need to be addressed to understand the influence of families on their adolescents’ health behaviors. In Qatar, among these factors are religion and culture. To understand the influence of Islam as a religion on family health values regarding physical activity, the Qur’an (the holy book of Islam) and the Hadith (the teachings of the Prophet Mohammed) are essential resources for committed Muslims.

Good health is considered a great blessing in Islam [13]. Islam emphasizes the development and maintenance of spiritual and physical strength regardless of gender [14]. Islam explicitly encourages Muslims to teach their children different sports in an attempt to make them strong and physically active. The Hadith says, “Teach your children swimming, throwing arrows and riding horses” [15].

Islam is not only a religion but also a belief system that influences the individual in his/her spiritual totality [16]. In Muslim countries, it is difficult to separate and differentiate religious values from culture. Religious values vary from one Muslim country to another, which reflects the diversity in religious practices. This cultural diversity arises from the different interpretations of the Qur’an and Hadith. Muslims have the general laws, or Sharia, which can be found in both the Qur’an and Hadith. In addition, there are different schools of thought (fiqh). Fiqh laws are “deduced” from sharia, which are specific and changeable according to circumstances in which they are applied [17]. Each school of fiqh provides a unique interpretation of the Qur’an and Hadith. However, Islamic countries differ in what school of fiqh they follow. Therefore, Muslim families from different regions will differ in their traditions and thus in their values regarding physical activity.

To better understand the various religious health values regarding physical activity, we will address culture as another context. Culture can be defined as “a set of characteristics, behaviors, rituals, and beliefs that are used to describe a group of people who: (a) live within (or originated from) a specific country or geographical region; (b) share a religious affiliation; (c) claim common ancestry and heritage; or (d) are grouped together for other reasons” [18].

### Theoretical perspectives: Bronfenbrenner’s ecological approach

To understand the influence of family on the physical activity of adolescents and young adults, we should be aware of the contexts and interrelationships between contexts in which this behavior occur. Bronfenbrenner’s ecological approach to the study of human development has focused on the interaction between the developing child and the immediate environment in which he or she lives and the influence of the broader context in which this interaction takes place [19]. These contexts, according to Bronfenbrenner, are microsystems, mesosystems, exosystems, and macrosystems. Applying these principles to understand the ecologies and contexts in which physical activity behavior is embedded will enable us to understand the complexity of the health behavior.

The microsystem of Bronfenbrenner’s ecological approach explains the physical activity of university students by addressing the family values as the context in which the activity exists. Previous research has focused on this context, ignoring other ecologies that may shape the health behavior of adolescents. Applying the exosystem will help explain the other contexts influencing family values. The exosystem focuses on the impact of external settings that have no direct effect on the adolescent, and it will help in examining family religious and cultural values regarding physical activity. Finally, the macrosystem explains another context that influences family health values and, as a consequence, adolescent physical activity, which is the Qatari culture. This ecological approach not only helped in reviewing the literature and framing the interview questions, but it also influenced the analysis process and findings’ interpretation.

Understanding the health and well-being of adolescents and young adults has been neglected, which presents a gap in the research about adolescent health [20]. Non-communicable diseases are considered a public health threat in Qatar and innovative strategies are needed to tackle them. This study will shed light on the important role that family plays in influencing physical activity among adolescents and lessen the burden of non-communicable diseases as a major objective of the National Public Health Strategy in Qatar. The goal of this study is to understand how adolescents’ physical activity can be shaped by sociocultural factors, particularly family health values and Muslim Arab culture.

## Methods

### Recruitment and participants

After the Institutional Review Board (IRB) at the university approved the research proposal, we began collecting the data. Flyers inviting participation were posted in different places on campus including male and female sections. The recruitment material explained the inclusion criteria, basic information about the study, and contact information for the first author. Snowball sampling was also followed by asking participants to share the study information with those they knew who might fit the criteria. In addition, the researchers shared the study purpose and related information with students in their classes and recruited some students.

Most of the interested students contacted the first author by email. If a student met all the criteria and agreed to participate, an interview would be scheduled. Data collection continued until we reached data saturation. Study participants were undergraduate students at the university. A criterion sampling strategy was used in the study. Criteria for study participation included undergraduate students aged from 18 to 23 years who are Qatari or Muslim students who were born and had been raised in the State of Qatar**.**

### Data collection

Students consented to participate in face-to-face, semi structured interviews, which were conducted on campus. The interview questions were written in English and then translated into Arabic (See Additional file 1 for the interview guide). The first author was responsible for conducting all the interviews. Participants were given a choice to use whatever language they liked when answering the questions. In the first part of the interview, participants were asked if they consider themselves active or not, and the types of activities they perform. The next set of questions was about family values regarding physical activity and what mostly shape these values. The final set of questions was to understand how the family health values, shaped by Islam and culture, influenced the participants’ physical activity. Follow-up questions were used to elicit more information and investigate. Each interview lasted approximately 45 min. The interviews were digitally recorded and transcribed verbatim. Participants were asked about the possibility of conducting additional interviews to clarify any issue raised from these interviews and most of them agreed.

### Data analyses

Inductive qualitative analyses involved discovering the themes in the interviews. Data were analyzed using constant comparative techniques. Pieces of data were compared for similarities and differences [21]. Each transcript was coded, and new themes were added to the codebook as they emerged. Constant comparisons were conducted to differentiate one theme from another and to identify dimensions of each theme. With each new addition of data, themes were added and modified as needed. Finally, the themes were combined into a coherent textural description of the phenomenon. The authors analyzed the first five interviews together, then they worked independently to analyze the data to help in the verification process. We read through the transcripts and identified the common themes separately, then came to a consensus regarding the themes and categories.

## Results

Study participants were 20 undergraduate students at the university (Female students =10, Male students = 10). The participants aged 18–23 years who were Qatari or were Muslim students who were born and had been raised in the State of Qatar. They have different nationalities including: Qatari, Syrian, Palestinian, Egyptian, Sudanese, Somalian, Bangladesh, Pakistani and Indian. (See Additional file 2 for a description of the sample; for confidentially and anonymity reasons, each student was assigned a number).

The majority of male participants felt that they were physically active; they exercised regularly, attended gym clubs, ran, walked, did track and played football. A few of them mentioned that they were working on improving their physical activity since they did not exercise regularly.

Female students differed in their physical activity behaviors. A few students attended gym centers at the university or off campus, attended centers to learn swimming, or had gym centers at home; as one student noted:

“On occasion, not all the time, I have to push myself. We have like a gym, not exactly a gym, but we have all the, all the... We have a small gym at home. I would go there just to exercise, but I always think about it as a burden, because you know sometimes although I do agree and acknowledge all the positive effects, sometimes just I have to push myself to do that.” (114)

Other participants did not exercise regularly, and the majority were not active at all. Some mentioned that they considered themselves active because they walked in the neighborhood and they did housework: “I am physically active; I work at my home, I just tiding rooms and so on. I mean because we walk around the compound, and I see what I do is enough because I get tired fast.” (107). One female student practiced basketball at the university without her parents’ knowledge: “Now, I also play in the basketball university team. Yeah, although my family does not know (laugh), I feel I am now 20 and I’m independent. I do exercise in my home, I run every day, but umm I don’t get support for that.” (115).

### Family health values regarding physical activity: the microsystem level

#### Male role models in physical activity

Half of the participants mentioned that their families were not active at the time of conducting this study. Almost all of the other participants who believed that their families were active showed that only fathers were physically active and motivating them to be active, and mothers were not active because they were “housewives” and thus did not socialize to be active. They explained that mothers and female siblings will give priority to housework, taking care of family members, or just spending a relaxing time over physical activity. One participant mentioned:

“So most housewives stay at home, which is not a good thing for their health. But my father is working in the military, so he has a good health activity in the military. He trains the personnel over there. So he has a good health aspect, but for the mother side not much because she does not go to a job and she just like cook or just sit at home and watch TV.” (102)

An Indian male student explained that his father is someone who is very “conservative about health”, he elaborated: “My father is the leader of the family, so we do whatever he says, whatever the way he chooses we follow, we just follow him.” But, he explained that his mother never cared about physical activity nor motivated them to be active.

A Sudanese female student talked about her father motivating her and other female siblings in the family to be active. She said that her father tend to buy sporting suits and shoes for everyone when planning to go exercise and encourage them in different ways. He also shared health promotion brochures with them regarding the importance of physical activity for their health. On the other side, her mother was satisfied to be active by doing house chores: “Ya, she always at home, she works a lot in the home so at night she will just relax and watch television.”

Participants also mentioned that male siblings were more active than females in their families. The majority mentioned that males would play football, ride horses, and exercise at gym centers. One female student explained: “Only fathers and brothers exercise and play football. For women in our family, we only think about exercising, but we never practice it.” (116). Another student mentioned that only her father goes to the gym to exercise, and if her mother and the other female siblings want to be active they could only walk.

Participants in our study showed that males will be motivated to do sports, swim, and join gym centers to be active. On the other side, females will be motivated to be active by walking because they want to socialize, have fun and enjoy their time. They also explained that females were not allowed to practice some sports, which may hurt them and felt that their parents were overprotective:

“My sister was really interested to do horse riding and swimming, but my mother refused. She was afraid that my sister might fall down or something bad might happen. That’s the reason why she refused. There are some aggressive sports, we are not allowed to practice them. She even refused me joining football and basketball team.” (112)

Only one female participant talked about her mother as a resource to motivating all the family members to be active. She explained:

“My mother used to take care of her health and fitness by exercising. Actually, until recently, she went to the gym and exercised. In our family, my mother tries to promote a healthy style, and she encourages us to walk and to be active*.*” (108)

#### A health condition will motivate families to be physically active

The majority of the participants showed that their families encouraged them to be active if they (the parents, other siblings and the participants) were overweight or obese and had a health issue such as diabetes or hypertension. These families were advised to be active by health care professionals. People with health issues would try to change the lifestyle of the other family members, thus enhance a support system of the surrounding environment. One student mentioned the following:

“Before my father had high blood pressure they didn’t care that much but nowadays their values towards health have become different because he always linking blood pressure with gaining weight, so he tried to be physically active and just taking us with him.” (107)

#### Families give priority to work and academic achievement over physical activity

Participants showed that their parents and siblings were not active because they spent a long time at work and school/university. This theme was clear among participants from immigrant families. As one participant explained: “Because no one has the time to go out or to be physically active, my father works like 10-15 hours so he does not have the time and my mother as well she is a house wife she does not go out much as well.” (106). A female participant mentioned that she wished her parents would value physical activity and motivate her to be active and do sports as they always encourage her to have better accomplishments in her studies.

Some participants reported in this study that parents believe adolescents or young adults do not need to be active, since they are still young and they do not suffer from any health problem. Participants also mentioned that siblings in their families might temporarily value physical activity to stay in shape, become muscular, and obtain a job.

The majority of male students perceived their family health values positively, and they believed they could be role models for other families, although some of them did not have clear health values of physical activity and were not active. Family health values shaped the physical activity of these participants. One participant, who described his father as active because he worked in the military and his mother sedentary because she was a homemaker, mentioned:

“For my family, they influence me positively. They have never influenced me in a negative way. They do their job properly so my family I consider it as a great family. They should be, they should act as a role model for other families in acting such healthy.” (102)

Female participants showed different perceptions toward their family health values regarding physical activity. A few of them showed positive perception that shaped their behaviors and encouraged them to be physically active; as one participant explained: “My mother affects my behavior a lot, as I told you before, my mother is taking care of our health and she encourages us to be active.” (108)

Other students perceived their unhealthy family values negatively, and as a result, they ended up being more active than their parents were; yet, we also saw others who ended being physically inactive like their parents. One participant said: “There is no motivation or encouragement. ‘It is not worth it’ this is what they said. They discourage me. However, I love sports, and I will practice it because this is coming from my inside; it is my inner feeling, and I love sports”. (110)

### Religion’s influence on family health values and adolescent physical activity: the exosystem level

#### Explicit influence of religion on family health values

A few participants discussed the explicit influence of religion on family health values of physical activity. Participant (# 102) said that his family would tell him: “Go and learn how to swim because our prophet encouraged us to learn swimming and riding horses.” These participants showed that religion is an important factor in shaping family health values regarding physical activity. They mentioned that their families believed that healthy behavior is what Islam asks them to follow: “Yes, for physical activity in Islam, my family believes that Islam actually tells you that you have to do these physical activities, you have to keep a good body shape or good health.” Some of them explained how Islamic practices such as praying, making ablution, and attending the masjid has a positive influence on human health; as one participant mentioned:

“I read about praying and some scientists prove that praying like 5 times a day and making ablution also, and how it has effects on the human body, and it refreshes the blood cycle inside the body and doing this five times a day at least, I think it affects to have healthy life or healthy body.” (101)

On the other hand the majority of the participants demonstrated an implicit influence of religion on their family health values regarding physical activity. However, the prophet’s teachings were included in their explanations when asked about the influence of religion on their family health values. One participant explained the following:

“I don’t think that it is directly influenced. Sometimes like my mother like encourages me to do some activities, like to go to swim, because I don’t like to swim, and learn how to swim because of our prophet, he encouraged us to learn swimming and riding horses, so like sometimes she tells me to do this, but I don’t like it.” (101)

#### Culture’s influence on family health values was veiling religion’s

When participants were asked about the influence of religion on their family health values regarding physical activity, the majority mentioned there is no influence. They meant that they were not motivated to be active because of religion. The reason they did not see religion influence as a separate entity is that it is hard to separate religion from culture in Arab Muslim countries, and culture influence is veiling religion and actually, it is the dominant factor in our study. One participant explained:

“May be my mother is more influenced by religion than me. My mother is influenced by religion and culture. But, we girls are influenced by culture more than religion. Not everyone here is following religion regarding physical activity. I do not think religion motivates me to be active. I wanted to play football and basketball, but my mother didn’t allow me and prevent me from joining the teams. ” (112)

These female participants were trying to explain that their families would encourage them to be active within the boundaries of Islam, thus dressing modestly and sex segregation were issues discussed by female participants that prevented them from being active. All of the female participants in this study wore hijab (veils) as a result the dress code of different sports was reported as a limiting factor for them from being active. They explained that it is still uncommon to were hijab and do sports, as one participant mentioned: “Like the hijab. Yeah its limiting. Yeah, I believe if I wasn’t wearing it, I would be now in…maybe a basketball team, Qatari basketball team. Yeah but if there are some places like only for females that’s fine and that’s good.” Another participant talked about socializing with males in public when practicing some sports is prohibited by the family and how it is a need to have female facilities. One student elaborated on this:

“Islam encourages us to swim, throw arrows, and ride horses, right??. Islam encourages sports, but that doesn’t mean to go… I can learn swimming, but keeping the Islamic boundaries. Islam does not allow us to socialize with males and go on public. No, this is not accepted by my family. NO” (109)

As a Qatari male participant explained: “Like my brother and I go to the gym. My sister, we have a gym at home so she uses the gym at home, because we do not have any gym like female gym nearby. So ya part of the family is physically active not the whole family is active.”

Female participants also showed that the Islamic context that was encouraging physical activity is absent in the Arab culture, as one student explained: “Islam promotes sport and health, where our prophet Muhammad said: (Teach your children swimming, archery, and horse riding). However, unfortunately, Arabs do not follow the instructions of Islam. If they did, all of us would be athletes.” (109). Thus, it would be more appropriate to address culture as the next factor influencing family health values regarding physical activity.

### Culture’s influence on family health values and adolescent physical activity behavior: the macrosystem level

#### Culture as a facilitator for males’ physical activity

Culture has a major role in shaping family health values regarding physical activity in our study. A few participants, mainly males, stated the positive role of Qatari culture in shaping their family values. One participant explained that as Qatari males, they were supposed to practice horse riding and the other traditional sports in Qatar, and they need to follow the steps of their ancestors and practiced the sports they used to do in their free time. Thus, culture was seen as a facilitator for physical activity from the males’ perspectives, which was not the case for female participants who reported the negative influence of culture on their physical activity.

#### Culture has a negative influence on females’ physical activity

Almost all female participants mentioned that culture had a negative influence on their physical activity because of the limited opportunities available for them compared to males in Qatar. The majority showed that they were not allowed to work out in parks or attend gym centers; as one participant mentioned:

“It is not accepted by our culture that a Qatari woman goes to exercise in a gym center with males. There should be one for women only, which you cannot find here or there are a few of them…Because I am a girl, I have limited choices. Although my country is paying more attention to physical activity, the culture has a stronger effect on our behavior.” (109)

Most females showed that they need to have permission and approval from their families to exercise and some families would be worried about their daughters being injured in sports: “The big issue is to get permission to work out. Girls are not allowed to leave home and come back whenever they want, NO NO. They are always scared that we may hurt ourselves.” (110) In addition, as with other women in many Arab cultures, they need to be accompanied by a male family member when going outdoor, which reduces the opportunity for physical activity. Female participants in our study also explained that having maids who did all the housework and drivers for transportation lead them to have a sedentary lifestyle:

“I think it is because we get a lot of help from the drivers like going to get food for us, we do not go out to get it, and we have maids cleaning, we don't clean you know we do not generally move in regular day. I mean, because a lot of help encourages us to be lazy.” (114)

#### Culture of origin is the dominant factor in shaping health values for immigrant families

Participants whose parents migrated to Qatar for better living and work conditions mentioned that their culture of origin played a major role in shaping family health values regarding physical activity. They showed variety in their health values and how their countries of origin influenced them, but most of these values were motivating them to be active. Participants noted that in their countries of origin, people were less dependent on transportation than in Qatar and did not have drivers or housemaids. Some explained that the influence of their culture of origin on their family health values was the dominant factor. As one participant explained: “You know the yoga kind of things, because I came from somewhere where yoga is very popular in our society, you know the village where I came from, so my father is someone who is very, I mean he cares a lot about these things.” (103) Even Qatari students in our study mentioned that cultural diversity in Qatar has a positive influence on the Qatari values regarding physical activity; as one participant mentioned, “People bring healthy values from their countries of origin to Qatar which motivate us to be physically active.” (102).

Male students from immigrant families who reported that their family health values were shaped by religion and their culture of origin perceived these values positively and were more likely to be physically active than those who were only influenced by Qatari culture. One Syrian student explained:

“I think like it’s my father’s family, mother’s family… they are all the same. Like the places they grew up in, the place they come from, it’s the same. When I go to my grandparents, it’s the same situation, they eat healthy foods, no smoking, they try to go out a lot and stay active, so I think they like inherited this one from their parents’ home country.” (101)

The majority of the participants mentioned that the concept of physical activity is new to Qatari culture and that cultural values regarding physical activity are changing for better in Qatar. One participant stated: “I think it is just a new thing eh I mean new behavior to them, they are not used to it. However, the new generation is different; they are trying to be active.” (104).

While addressing culture as a factor, the responses showed a clear intersection with the physical environment and the country policy toward enhancing physical activity in Qatar. Participants pointed to the role of the physical environment and the state in supporting physical activity, such as the availability of sidewalks and parks and the designation of a sport day: “Here in Qatar, they have like a lot of new parks, a lot of sidewalks to encourage people to go outside, walk and have some activities.” (101).

## Discussion

Previous research has focused mainly on individual factors, rather than on the influence of sociocultural factors and different surrounding ecologies on adolescent and young adult behaviors. In this study, we address the family context to understand physical activity behaviors among university students. Understanding family health values of physical activity and the factors shaping these values, such as Islam and culture, helps explain the behaviors and create a plan for effective health promotion interventions and programs that may enhance active lifestyle for young adults.

### Family health values regarding physical activity

Previous research has found that family environment plays an important role in the physical activity of Arabic adults [22]. The majority of students in our study who perceived their families’ values positively were more likely to be influenced by their values. Family health values reported by the majority of the participants showed male role modeling of physical activity with fathers and male siblings were more likely to be active than their mothers and female siblings who did not value physical activity. A few reported that both parents valued physical activity and thus both genders in the family were active. Consequently, participants’ physical activity was influenced by these values. The majority of male participants were physically active; they exercised regularly, attended gym clubs, ran, walked, did track and played football. In contrast, females were active by walking and doing housework because they want to socialize, have fun and enjoy their time. Only a few female students attended gym centers, and the majority did not exercise regularly and were not active.

Aligning with our findings, Ramanthan and Crocker [23] reported that participants, regardless gender, were taught to play sports by their brothers, engaged with physical activities with brothers and fathers, and were motivated to be active by males in their families. They found that although the primary role models were males, fathers and brothers were more likely to promote fun-based environment. They explained that fathers and brothers will not find themselves racing daughters and sisters; yet, they will focus on enjoying their time as a family.

Some students mentioned that their families will give priority to work, family commitments, and academic achievement over physical activity. They explained that their families were not active because the father would spend long hours at work and the mother was taking care of family members and doing housework, which negatively impact physical activity time and choices. Previous research supports these findings; Kahan [24] found that a major barrier to physical activity among women was having family commitments such as household chores and child care that were prioritized over exercising. Once the participants perceived these values positively, their physical activity behavior was negatively influenced. Male participants of these families reported that they were spending time at home around the family members who were busy and did not have time to exercise, and female students reported that they were active by helping with household chores. Taylor and Toohey [25] explained that parents often felt that physical activity is not appropriate for girls and prioritized academic achievement over it, which made females feel that they need to devote free time for school work, family, and domestic role. The public in Qatar needs to be educated that participation in healthy lifestyle would benefit the individual and the family, which might be a culturally-tailored message that promote active living among women in Qatar [8].

A major family health value reported by the participants was that families were not concerned about being physically active unless they had a health issue. Prior research supports this finding; Benjamin and his colleagues [26] reported that the presence of a health condition was a motivator for individuals to exercise. These values influenced the participants’ physical activity in our study. Therefore, when one of the parents or a sibling was overweight/obese or had a health condition such as diabetes or hypertension, they would likely start to exercise and encourage others to be active. Otherwise, they would not support physical activity because they were young and healthy—at least, according to the participants’ explanations. According to WHO [2], physical activity has significant health benefits and contributes to prevent noncommunicable disease. Thus, the crucial role of physical activity in preventing noncommunicable diseases, and not just alleviate them should be communicated clearly to the public.

### Religion’s influence on family health values and adolescent physical activity

To understand what shaped these family health values, we addressed other contexts: culture and religion. The exosystem in the ecological model helped us examine family religious values regarding physical activity. Both the Quran and the Hadith of Prophet Muhammad provided verbal modeling in which values are conveyed, shaping behaviors and celebrating community cooperation and comfort from the challenges of daily life [27]. Religion was cited as a crucial factor in motivating Muslims to be active and follow healthier lifestyles [28]. Similarly, a few participants in our study noted that there was an explicit, positive influence of Islam on their family values of physical activity. These participant were more likely to be active than others, although they did not see the direct influence of religion on their physical activity behaviors. In their explanation, they pointed to the Prophet’s teachings about the importance of physical activity for Muslims.

Female participants perceived the influence of religion positively on their physical activity behavior. Families were motivating them to be active; yet, within the boundaries of their religion. Girls are not allowed to exercise in parks or in gyms unless such spaces are segregated by sex. Thus, dressing modestly and sex segregation in recreation facilities were issues reported by female participants that prevented them from being active. Previous studies reported that this is related to religion and public modesty [29]. Nakamura reported the same issues that prevented Muslim females from participating in some sports in Canada and mentioned that Islam should not be reported as a barrier to female physical activity [30]. The author elaborated that actually the dress code for some sports and the lack of facilities that meet the needs of these females, were the reasons that might prohibit them from being active and encourage sedentary lifestyle. A study reported that Qatari adolescents are more likely to be inactive due to lack of suitable places and or facilities for physical activity [31]. Donnelly and Al-Thani [8] recommended that modesty and religious practices should be considered by policy makers and planners when designing facilities for women in Qatar.

In our study female participants wanted to make it clear that the Islamic context that would encourage them to be active, was absent from the surrounding Arabic culture. They also wanted to point that the dominant factor in shaping their family health values regarding physical activity and their physical activity behaviors was mainly culture. If the Islamic context was not emphasized, physical activity especially for females will be quoted as prohibited [30]; “Individuals should be free to curve out a comfortable space for themselves, one which conforms to the customs of the adopted culture, but only because it is meaningful within the context of Islam.” (p. 32).

### Culture’s influence on family health values and adolescent physical activity

Anthropological and sociological studies of physical activity have long recognized the central role culture plays in influencing adolescents’ perceptions and participation in physical activity [9, 23, 32–35]. Within Arabic culture, which is a patriarchal culture, gender raises a range of considerations. Male participants talked about the positive influence of Qatari culture on their physical activity, which encouraged them to be active by making different sports available for them. Qatari males were motivated to practice traditional spots promoted by Qatari culture such as swimming and horse riding. They would practice activities followed by their ancestors in their free time. Following these activities will help them construct their identities and enrich the sense of belonging to the culture.

On the contrary, female students mainly showed the negative influence of Qatari culture on their physical activity, given the limited choices available to them. As for their families’ inactive lifestyle and unclear values regarding physical activity, female students singled out Qatari culture as the main barrier to physical activity. Female participants mentioned that one of the cultural issues that might prohibit them from being active was the need for having a permission and approval from their families to exercise. This notion was supported by previous research indicating that women in some conservative Islamic countries are not allowed to socialize without being joined by a male family member, which may decrease the possibility of exercising [30]. Participants in our study also pointed that they were not allowed to practice various sports, but they were encouraged to walk or do house work to stay active. They explained that there was a fear that they might hurt themselves or took on “masculine characteristics”. Diaman [36] called this the fear of “defeminization”, which showed that women who try to be active may become physically strong and adopt male characteristics.

Currently, some Muslims are undergoing through many cultural changes due to the modernization of economies, whereas others remain less affected by these factors. In the culture of modernized economies, there is an emphasis on individual fulfillment. As a result, more women are joining the work force and launching careers. Women in countries with modernized economies are increasingly participating in sports. In countries with less-developed economies, these activities are seen as unnecessarily risky and frivolous in comparison to the need for taking care of children and the home. In general, single life for women in these societies is economically unsustainable, and sporting activities are felt to not fit into a woman’s life.

Thus, a Muslim in such a society tends to think of women participating in sports as ‘un-Islamic’, while a ‘modern’ Muslim thinks of such participation as Islamic and the prohibition of women from sports as un-Islamic. These differences are perceived to be based on culture, not religion [27]. Therefore, there is often not a clear answer as to whether something is Islamic or not, which applies across all situations. Often, modernized Muslims have the illusion that they can clearly distinguish between culture and religion because modernized people are often blind to the fact that they also have a culture. Usually, what someone thinks is ‘pure Islam’ without any culture mixed in is actually just Islam as lived in the context of modern culture. In such a case, ways of thinking about women’s roles in society, there is a fear of what other people think of such a ‘sporty’ girl, may move people to think of women playing sports as ‘un-Islamic’. However, for modernized people, it is not so, for they are not under the scrutiny of a close-knit society. Nevertheless, Qataris now live more sedentary lifestyles, so the need for exercise is greater than before, and sports play a useful role in realizing an Islamic value—good health—which it would not have played previously.

Another cultural issue reported by the participants was the dependent on house cleaners or housemaids for doing house work and drivers for transportation. This made them feel lazy and enhance their sedentary life style. This was reported as a barrier for physical activity and women in Qatar recommended doing more housework and being less dependent on these maids [12].

An interesting finding from our study is that non-Qatari students revealed that their family’s culture of origin was the dominant factor in shaping their family health values. These families still had the same values of physical activity from home countries, such as yoga for men, and walking and housework for women. These immigrants wanted to emphasize their cultural traditions from their countries of origin to keep their identity and feel the sense of belonging.

The majority of non-Qatari male students in the study recognized these values as healthy, yet it was not the case for non-Qatari females, who had the same complaints as Qatari female students because of their limited options. Generally, non-Qatari students were more active than their Qatari counterparts. These students were influenced by the values of physical activity of their family’s culture of origin; they practiced sports from home country, were less dependent on drivers and house workers, they walked more and shopped by themselves, and did housework.

Finally, the majority of the participants showed that active living is a new phenomenon to the Qatari culture, which is a behavior that individuals did not get used to follow. They also pointed to the great efforts that the government in Qatar is putting to incorporate physical activity in the lifestyle of all individuals and they were optimistic that the new generations will adopt physical activity as a way of life. Donnelly and Al-Thani [8] also recognized the role of the government in implementing various strategies to promote active living for men, women and children.

## Conclusion

This type of research helps understand the complexity of physical activity behavior by addressing the different sociocultural factors shaping it. The shift toward social determinants of health among young people means that the major threats to their health and well-being are increasingly rooted in the organization and expectations of everyday life [37].

This study emphasizes the specific roles of family values, culture, and religion in shaping adolescents’ perception of and behavior regarding physical activity. In this setting, male participants were more likely to report being physically active. In contrast, female participants were more likely to report decreased value on physical activity due to cultural influence.

The findings of our study can not be generalized due to the selective and nonrepresentative nature of the sample. Recruiting by snowball sampling technique made us interviewed students who were suggested by other participants and who, in some cases, were friends. These participants, because they were friends, might have similar religious and cultural values and that may have limited the diversity we were looking for among the participants. Findings and conclusions of the study should be treated as hypotheses for future testing rather than as definitive. In addition, having the interviews conducted by a professor from the university may have risen the potential for social acceptability biases. To lessen the effect of such biases and while conducting the interviews, transcribing, and then during the analysis and interpretation processes we attempted to remain clear and open minded. We were ready to learn from these students about the ecologies shaping their physical activity behaviors. Our religious and cultural values were put aside and were open to any new experience and nothing was determined in advance.

The findings address gaps in the literature about families’ health values regarding physical activity in Qatar, the influence of the different ecologies surrounding these values, and the physical activity behaviors of college students. Future research should be conducted to better understand the parenting practices that families follow in sharing health values regarding physical activity with their adolescents because this may influence adolescents’ perception of these values and behavior. In addition, more research is needed to understand the influence of peers and media on physical activity. Finally, more context-specific research addressing the family context and other different ecologies is crucial to understanding different health behaviors for adolescents, such as eating behaviors and drug use, in order to plan for evidence-based health promotion interventions.

The findings will guide public health specialists, health educators, social workers and other professionals working with adolescents and young adults to promote their health. Participants in this study showed that Qatari culture is family centered. Knowledge about these factors can aid in the development of family-based interventions designed to motivate adolescents to be physically active; these interventions should be religion- and culture-tailored.

Professionals should work with children and adolescents, regardless of their gender, to enhance their physical activity behavior and thus spread the role modeling to female figures in the family. Health educators and promotors need to emphasize the role of physical activity in prevention of chronic diseases, in addition to managing them. In addition, support groups can be conducted with working parents to aid in managing their schedule and make them spare some time for physical activity.

Professionals should address the Islamic context as beliefs and practices to communicate messages that promote physical activity among Qatari people Adolescents should be aware of the religious values and the prophet’s teachings regarding physical activity, which may shape their attitudes and active living. The importance of religious practices, such as praying five times a day can be communicated by religious leaders to enhance physical and spiritual health. Professionals should respect females’ needs in Arabic Islamic countries and advocate for women-specific facilities to encourage them be physically active.

Messages communicated to young adults to enhance their physical activity behavior have to be contextualized and culture-tailored. Professionals should consider incorporating the traditional sports for Qatari ancestors in any physical activity program, work with young adults to increase their level of participation in house chores and be less dependent on drivers, and respect the cultural diversity in Qatar while working with young adults from immigrant families to enhance their active living. These individuals may have various family health values regarding physical activity, which they adopt from their countries of origin. Thus, professionals should consider including activities that are common in countries of origin of these young adults when promoting their physical activity behavior.

## Supplementary information

**Additional file 1.** Interview Guide.

**Additional file 2: Table S1.** Demographics for Participants from Qatar University.

## Data Availability

The datasets used and analyzed during the current study are available from the corresponding author on reasonable request.

## Abbreviations

GCC: Gulf Corporation Council
IRB: Institutional Review Board
WHO: World Health Organization
