# Supplementary material for: Addressing context to understand physical activity among Muslim university students: the role of gender, family, and culture
Source: BMC Public Health. 2019 Nov 5;19:1452. doi: 10.1186/s12889-019-7670-8 (PMC6829810; doi:10.1186/s12889-019-7670-8)
Supplement: Supplementary file 1 — Additional file 1. Interview Guide. [file 12889_2019_7670_MOESM1_ESM.docx]

Additional file 1: Interview Guide.

1. Who is a physically active person? Do you think you are physically active? What do you practice to be active?
2. How do you feel about your family health values regarding physical activity?
3. What do you think that mostly shape or influence your family health values regarding physical activity?
4. What do you think about the influence of Islam on your family health values?

- How are family health values influenced by Islamic beliefs?
- How are family health values influenced by religious practices?

1. How do you feel about the influence of culture on your family health values?
2. What do you think about your family influence on your health behavior?
3. What do you think about religion influence on your health behavior?

- Do religious injunctions had an impact on your decision whether to practice physical activities .i.e. walking, running..etc or not?
- Are you aware of these injunctions? Or are you aware that Islam concerned with the development and maintenance of physical activity?
- If you became aware of such religious injunctions, suggestions and recommendations, would that change your attitude towards practicing physical activities?

1. What do you think about culture influence on your health behavior?

Demographics:

Male/Female:

Age:

Nationality:

Major/year:
