# Supplementary material for: Addressing context to understand physical activity among Muslim university students: the role of gender, family, and culture
Source: BMC Public Health. 2019 Nov 5;19:1452. doi: 10.1186/s12889-019-7670-8 (PMC6829810; doi:10.1186/s12889-019-7670-8)
Supplement: Supplementary file 2 — Additional file 2: Table S1. Demographics for Participants from Qatar University. [file 12889_2019_7670_MOESM2_ESM.docx]

Additional file 2: Table S1. Demographics for Participants from Qatar University

| Participant # | Age | Gender | Nationality | Major |
| --- | --- | --- | --- | --- |
| 101 | 22 | Male | Syrian | Computer Science |
| 102 | 20 | Male | Qatari | Management |
| 103 | 23 | Male | Indian | Management |
| 104 | 20 | Male | Pakistani | Environmental Sci |
| 105 | 20 | Male | Pakistani | Environmental Sci |
| 106 | 21 | Male | Bangladesh | Environmental Sci |
| 107 | 23 | Female | Sudanese | Health Education |
| 108 | 21 | Female | Qatari | Media |
| 109 | 20 | Female | Qatari | Education |
| 110 | 23 | Female | Palestinian | Media |
| 111  112  113  114  115  116  117  118  119  120 | 23  22  18  20  20  20  20  21  22  23 | Female  Female  Female  Female  Female  Female  Male  Male  Male  Male | Qatari  Qatari  Omani  Qatari  Qatari  Qatari  Qatari  Qatari  Qatari  Qatari | Health Education Media  General Arts Biology  Biology  Biology  Business  Business  Engineering  Engineering |
